# Association between CMV and Invasive Fungal Infections After Autologous Stem Cell Transplant in Lymphoproliferative Malignancies: Opportunistic Partnership or Cause-Effect Relationship?

**DOI:** 10.3390/ijms20061373

**Published:** 2019-03-19

**Authors:** Francesco Marchesi, Fulvia Pimpinelli, Enea Gino Di Domenico, Daniela Renzi, Maria Teresa Gallo, Giulia Regazzo, Maria Giulia Rizzo, Svitlana Gumenyuk, Luigi Toma, Mirella Marino, Iole Cordone, Maria Cantonetti, Anna Marina Liberati, Marco Montanaro, Anna Ceribelli, Grazia Prignano, Francesca Palombi, Atelda Romano, Elena Papa, Francesco Pisani, Antonio Spadea, William Arcese, Fabrizio Ensoli, Andrea Mengarelli

**Affiliations:** 1 Hematology and Stem Cell Transplant Unit, IRCCS Regina Elena National Cancer Institute, Via Elio Chianesi, 53 00144 Rome, Italy; daniela.renzi@ifo.gov.it (D.R.); svitlana.gumenyuk@ifo.gov.it (S.G.); francesca.palombi@ifo.gov.it (F.P.); atelda.romano@ifo.gov.it (A.R.); ematologia@ifo.gov.it (E.P.); francesco.pisani@ifo.gov.it (F.P.); antonio.spadea@ifo.gov.it (A.S.); andrea.mengarelli@ifo.gov.it (A.M.); 2 Molecular Virology, Pathology and Microbiology, IRCCS San Gallicano Dermatological Institute, Via Elio Chianesi, 53 00144 Rome, Italy; fulvia.pimpinelli@ifo.gov.it (F.P.); enea.didomenico@ifo.gov.it (E.G.D.D.); mariateresa.gallo@ifo.gov.it (M.T.G.); grazia.prignano@ifo.gov.it (G.P.); fabrizio.ensoli@ifo.gov.it (F.E.); 3 Genomic and Epigenetic Unit, Translational Research Area, IRCCS Regina Elena National Cancer Institute, Via Elio Chianesi, 53 00144 Rome, Italy; giulia.regazzo@ifo.gov.it (G.R.); maria.rizzo@ifo.gov.it (M.G.R.); 4 Infectious Disease Consultant, IRCCS Regina Elena National Cancer Institute, Via Elio Chianesi, 53 00144 Rome, Italy; luigi.toma@ifo.gov.it; 5 Pathology Unit, IRCCS Regina Elena National Cancer Institute, Via Elio Chianesi, 53 00144 Rome, Italy; mirella.marino@ifo.gov.it; 6 Clinical Pathology Unit, IRCCS Regina Elena National Cancer Institute, Via Elio Chianesi, 53 00144 Rome, Italy; iole.cordone@ifo.gov.it; 7 Hematology Unit, Tor Vergata University Hospital, Viale Oxford, 81 00133 Rome, Italy; cantonetti@med.uniroma2.it (M.C.); william.arcese@ptvonline.it (W.A.); 8 Oncology-Hematology, Santa Maria Hospital, University of Perugia, Viale Tristano di Joannuccio, 05100 Terni, Italy; marina.liberati@unipg.it; 9 Hematology, Belcolle Hospital, Str. Sammartinese, 01100 Viterbo, Italy; mmontanaro51@gmail.com; 10 Medical Oncology, San Camillo de Lellis Hospital, Viale Kennedy, 02100 Rieti, Italy; oncologia@asl.rieti.it

**Keywords:** CMV infection, invasive fungal disease, gram positive bacteria, gram negative bacteria, skin commensals, autologous stem cell transplant, multiple myeloma, lymphoma

## Abstract

Unlike allogeneic transplant, autologous stem cell transplantation (ASCT) represents a procedure with a low-risk of cytomegalovirus (CMV) symptomatic reactivation-infection/end-organ disease (CMV complications) and invasive fungal disease (IFD). However, novel drugs for the treatment of lymphoproliferative malignancies could cause an increase of such opportunistic infections, even after ASCT. To the best of our knowledge, there are no published data demonstrating an association between CMV and IFD in the autologous setting, while this association has been widely reported in allogeneic transplantation. We have reviewed our series of 347 ASCT in myeloma and lymphoma patients performed over a period of 14 years with the aim of investigating the descriptive and analytical epidemiology of bacterial, CMV and IFD complications, focusing on the association between CMV and IFD. Patients with myeloma have significantly fewer bacterial infections and IFD than patients with lymphoma, but a similar rate of CMV complications. Descriptive epidemiological data are consistent with the literature, indicating an overall incidence of 36%, 3.5% and 15.5% for bacterial infections, IFD and CMV complications, with a case mortality rate of 4%, 16.7% and 3.7%, respectively. A strong correlation between CMV and IFD exists, with 8 cases of IFD out of a total of 12 presenting a CMV complication. At multivariate analysis, a diagnosis of lymphoma, ≥3 previous treatment lines and age ≥60 years were found to be independent risk factors for IFD. Duration of neutropenia (ANC < 500/mm^3^) ≥7 days represents an independent risk factor for CMV complications, where neutropenia most likely represents a crude surrogate biomarker indicating a deeper and longer state of overall immunosuppression. From our data we conclude that (1) myeloma patients are at lower risk of bacterial infections and IFD as compared with lymphoma patients but are at equal risk of CMV complications, most likely as a consequence of a selective impact of bortezomib on Herpes Viruses infection control; (2) a significant association exists between CMV and IFD, although a possible cause-effect relationship remains to be determined; (3) IFD is a rare complication after ASCT but burdened by a mortality rate of about 17%, with peak rates in older lymphoma patients who underwent more intensive therapeutic regimens.

## 1. Introduction

Even in the current era of novel drugs and targeted therapeutic approaches, autologous hematopoietic stem cell transplant (ASCT) remains a backbone in the therapeutic route of most patients with lymphoma and multiple myeloma. ASCT has always been considered a procedure at low risk of cytomegalovirus (CMV) infection and invasive fungal disease (IFD). However, in a near future these opportunistic infections might become a significant challenge for clinicians dealing with lymphoproliferative malignancies as a consequence of the introduction of novel therapeutic agents (i.e.,idelalisib, bortezomib, fludarabine, bendamustine) with a powerful immunosuppressive impact [1,2]. Many studies confirmed that CMV reactivation represents a relevant complication also in settings other than allogeneic stem cell transplant [3,4,5,6,7,8,9,10,11,12,13]. As for IFD, most previously published studies in patients auto-grafted for the treatment of lymphoproliferative malignancies reported a low incidence ranging from 0 to 8%, though with a relevant mortality rate [14,15,16,17,18,19]. The putative relationship between CMV and IFD is still far from being clarified and the most relevant published studies have been performed in patients undergoing allogeneic stem cell transplant. Some of these studies showed that a diagnosis of CMV symptomatic reactivation/end-organ disease might be associated with a significant increase of IFD risk [20,21,22]. To the best of our knowledge, studies aimed at investigating the relationship between CMV and IFD after ASCT are still lacking. In this article we present data on the incidence and risk factors for such infectious complications in a cohort of 347 ASCT performed in myeloma and lymphoma patients between May 2004 and April 2018 in our Institute, focusing on the relationship between IFD and CMV symptomatic reactivation/end-organ disease.

## 2. Results

Patient characteristics at transplant are summarized in Table 1. About one third of transplants were performed in patients aged 60 years or older and 10% in heavily pre-treated subjects (≥3 chemotherapeutic lines). In case of tandem ASCT, the second transplant was excluded from this analysis. Almost all myeloma patients had undergone a bortezomib-based treatment before ASCT since 2011, whereas before this date they received a standard anthracyclines-based chemotherapy. As detailed in Table 2, a comparison between lymphoma and myeloma patients showed a significantly higher rate of bacterial (42.5% vs. 28%, *p* = 0.005) and fungal (5.3% vs 1.2%, *p* = 0.035) infections in the former, whereas the rate of CMV symptomatic infection/end-organ disease was not significantly different (16% vs. 15%). Bacterial infections occurred earlier, as compared with IFD and CMV symptomatic reactivation/end-organ disease (7 vs. 14 vs. 34 days, respectively; *p* < 0.001), involving either Gram-positive skin commensals (i.e., *Staphylococcus aureus*) or, more frequently, Gram-negative bacteria (i.e., *Escherichia coli*). A total of 12 cases of IFD were diagnosed with a crude overall incidence of 3.5%. Eight cases were classified as probable pulmonary aspergillosis and four were diagnosed as candidemia. As for CMV symptomatic infection/end-organ disease, a total of 54 cases were observed (15.5%). Bacterial infections were the most frequent cause of death (six cases), followed by both CMV (two cases) and fungal infections (two cases). Overall, only one patient died from a non-infectious cause. The overall case fatality rate was of 4%, 3.7% and 16.7% for bacterial infections, symptomatic CMV reactivation and IFD, respectively. Only a trend toward a higher transplant-related mortality (TRM) was observed in lymphoma as compared to myeloma patients (*p* = 0.066).

Clinical and laboratory parameters and outcomes of 54 cases of CMV reactivation requiring specific antiviral treatment are detailed in Table 3. No significant differences among lymphoma and myeloma patients were found. All symptomatic reactivation events were observed in patients presenting with CMV IgG before transplantation, with 34 days median time from transplant to first detection. However, only 5 cases of end-organ disease were diagnosed. Of them, three were classified as interstitial pneumonia and two as hemorrhagic colitis (the overall incidence of end-organ disease was 1.4%; the incidence of end-organ disease among cases of clinically relevant CMV reactivation was 9.2%). Table 4 and Table 5 show univariate and multivariate analysis, respectively, of putative risk factors for fungal, bacterial and CMV infections. A diagnosis of lymphoma, a refractory disease status at transplant and a longer duration of neutropenia (defined as ≥7 days with neutrophils count lower than 500/mm^3^) were all significantly associated with the risk of post-transplant bacterial infections (*p* = 0.005, *p* = 0.04 and *p* < 0.001, respectively). However, at multivariate analysis, only a neutropenia persistent for more than 7 days was significantly associated with the risk of bacterial infections (OR 2.16, 95%CI 1.29–3.74; *p* = 0.006; Table 5).

Regarding fungal infections, a diagnosis of lymphoma (*p* = 0.035), three or more previous treatment lines (*p* = 0.009) and an age over 60 years (*p* = 0.001) were identified as potential risk factors at univariate analysis. Multivariate analysis confirmed an independent role of all these risk factors in favoring fungal infections (OR 4.09, 95%CI 1.2–16.23; *p* = 0.039; OR 2.91, 95%CI 1.29–6.55; *p* = 0.012; OR 10.34, 95%CI 2.55–40.11; *p* = 0.001, respectively; Table 5). Finally, a refractory disease status at transplant and a longer duration of neutropenia were the two variables significantly associated with the risk of post-transplant CMV symptomatic reactivation/end-organ disease (*p* = 0.039 and *p* = 0.004, respectively). However, at multivariate analysis, only a longer duration of neutropenia conserved its significant association with the occurrence of CMV symptomatic reactivation/end-organ disease (OR 2.4; 95%CI 1.2–4.9; *p* = 0.009; Table 5). Results of a sub-analysis including only lymphoma patients are summarized in Table 6. The duration of neutropenia was found to be the only factor significantly associated with bacterial infections (*p* = 0.022). Conversely, the diagnosis (T-cell lymphoma > B-cell lymphoma > Hodgkin’s lymphoma) was the only factor significantly associated with CMV infection (*p* = 0.028), whereas more than three previous treatment lines and an age older than 60 years were the two factors significantly associated with the occurrence of fungal infections both at univariate (*p* = 0.031 and *p* = 0.002, respectively) and multivariate analysis (OR 4.53; 95%CI 1.1–18.53; *p* = 0.036; OR 7.58; 95%CI 1.83–31.7; *p* = 0.005, respectively; Table 6). A strong correlation between IFD and CMV symptomatic infection/end-organ disease was observed. In particular, in 8 (66.7%) of the 12 cases of IFD a simultaneous or more often subsequent symptomatic CMV reactivation occurred (Pearson chi-square value: 24.9; continuity correction: 21.01; *p* < 0.0001).

## 3. Discussion

This study has been performed with the aim to evaluate the overall incidence and risk factors for bacterial infections, IFD and CMV symptomatic reactivation/end-organ disease in a large cohort of patients with lymphoma and myeloma undergoing ASCT. Further, it was aimed at exploring whether an association between CMV and IFD may exist. The results confirm previous descriptive and analytical epidemiology observations and suggest some novel items to take into consideration.

### 3.1. Discussion on Descriptive Epidemiology Data

Consistently with the literature, our data indicate that bacterial infections, in particular gram-negative bloodstream infections, are the most frequent infectious complications occurring in hematologic patients undergoing ASCT and the first cause of mortality among these patients [23,24,25]. Secondly, our data show that the overall incidence of post-transplant CMV symptomatic infection/end-organ disease, using a clinically driven diagnostic approach without prospective surveillance of CMV DNAemia by PCR, is about 15%, with an incidence of end-organ disease of about 1% and a direct case fatality rate of 3.7%, with the majority of episodes easily managed through an outpatient clinical approach, according to previous published reports [3,4,5,6,7,8,9,10,11,12,13]. However, it is worth underlining that CMV reactivation rate could be underestimated using this diagnostic approach, mainly in cases without severe clinical presentation. Thirdly, we report 12 cases of IFD (eight cases of probable pulmonary aspergillosis and four cases of candidemia) among 347 patients, with an overall incidence of about 3.5% and a case fatality rate of about 17%. Once again, these data on IFD are similar to those reported in previously published studies [14,15,16,17,18,19,26]. It seems important to underline that in our study all cases of probable aspergillosis had typical radiologic criteria according to the European Organization for Research and Treatment of Cancer/Invasive Fungal Infections Cooperative Group and the National Institute of Allergy and Infectious Mycoses Study Group (EORTC/MSG). However, even though these criteria permit to standardize IFD for clinical trials purposes, they are not universally applicable to clinical practice. Typical radiological signs of IFD are detected almost only in neutropenic patients, whereas they can be difficult to detect in out-patient setting of lymphoproliferative diseases, where it could be possible to diagnose some cases of pulmonary aspergillosis also in case of atypical radiological signs (i.e.,“tree in bud”/micronodules or interstitial infiltrates). Interestingly, our data indicate that whereas myeloma patients are significantly less susceptible to bacterial and fungal infections, as compared to lymphoma patients, most likely because they are less heavily treated at the time of ASCT, nevertheless, their susceptibility to CMV symptomatic reactivation/end-organ disease is similar to that of lymphoma patients, confirming the fact that bortezomib, used for first induction in myeloma patients, might represent a specific risk for the reactivation of the Herpes Viruses [9,10].

### 3.2. Discussion on Analytical Epidemiology Data (Risk Factors for Infectious Complications)

Looking at the whole population of myeloma and lymphoma patients, the only factor independently associated with CMV complications is a longer duration of neutropenia (more than seven days), where neutropenia most likely represents a crude surrogate biomarker of a deeper and longer state of immunosuppression. Focusing the attention only on lymphoma patients, a diagnosis of T-cell lymphoma, as compared with B-cell and Hodgkin’s lymphoma, respectively, represents a significant risk factor for post-transplant CMV reactivation, as we previously reported [11]. Both these findings point to the pivotal role of cell-mediated immunity in the effective control of CMV infection. As for IFD, our data show that patients with lymphoma, an age of 60 years or older and previous administration of three or more treatment lines are at higher risk of IFD at multivariate analysis. These factors have been also highlighted in other previously published studies in which a previous IFD, three or more therapy lines, a prolonged neutropenia, a colonization by *Candida* spp., a corticosteroid therapy and a previous treatment with fludarabine were all recognized as risk factors for fungal infections in hematologic patients undergoing ASCT [2]. A universal mold-active prophylaxis is generally not recommended in the autologous setting, since patients undergoing ASCT are globally considered at low risk of fungal infections. However, our data suggest that lymphoma patients older than 60 years and undergoing ASCT after three or more previous chemotherapeutic lines should be considered at higher risk of IFD, and perhaps they deserve a mold-active prophylaxis. The prolonged neutropenia (defined as ≥7 days with neutrophils below 500/mm^3^) does not appear to be a relevant factor favoring IFD in our cohort. This data might appear in contrast with those reported by others, but, if we carefully analyze the previous studies, we can notice that the definition of “prolonged neutropenia” is not homogeneous and more frequently categorized as ≥14 days with neutrophils under the 500/mm^3^ threshold. Thus, the lack of a predictive value of prolonged neutropenia found in our study could be, at least in part, explained by the different approaches utilized to classify neutropenia. Further, differently from what Gil and colleagues reported [16], a previous treatment with Rituximab does not appear to represent a clear risk factor for IFD, though we have not evaluated the potential impact of fludarabine considering the very low number of patients that received this treatment before transplant.

### 3.3. Discussion on Novelty Items

Results from this study reveal, for the first time, a strong relationship between CMV symptomatic reactivation/end-organ disease and IFD in patients with lymphoproliferative malignancies undergoing ASCT. Our data clearly show a significant association between these two post-transplant complications considering that a simultaneous or subsequent symptomatic CMV reactivation occurred in about 67% of IFD cases. Several studies have been published in recent years about this association in allogeneic hematopoietic stem cell transplant recipients. Even though some of these studies failed to demonstrate a significant association between CMV and IFD [20,21,27,28,29], most of them reported a significant correlation between these two complications, being a post-transplant CMV reactivation associated to an increased risk of IFD [30,31,32,33,34,35,36,37]. However, the exact mechanisms responsible for this association are poorly understood, as yet and more extensive studies are needed to further explore CMV effects on the immune system and its impact on IFD. Yong and colleagues recently reported that a CMV reactivation, as detected by a prospective monitoring of DNAemia, can precede an IFD and should therefore be considered as a risk-factor for a late fungal infection complication [22]. The authors speculated about the exact mechanism leading to the increased risk of IFD, highlighting the potential role of ganciclovir-inducted neutropenia [38] and the immunosuppressive effects of CMV itself, sustained by the impairment of macrophage migration and antigen presentation [39,40,41]. In contrast, in our study the diagnosis of IFD occurred significantly earlier than that of CMV symptomatic reactivation in the majority of cases. There are at least two reasons which may explain this difference. First, in our cohort we did not perform a prospective monitoring of CMV DNAemia by PCR, according to our policy based on a clinically driven diagnostic approach (as detailed in Material and Methods section), leading to a potential delay in identification of CMV reactivation; second, in the study of Yong and colleagues the overall time from transplant to IFD diagnosis was of 76 days in all patients and of 184 days in patients experienced a CMV reactivation, significantly different to that reported in our study (14 days), being a late IFD occurrence a most unlike event in autologous setting. Taken together, our data indicate a mere association between CMV and IFD, a sort of partnership, although, at present, it is not possible to ascertain whether a cause-effect relationship exists. Are CMV infection and IFD two faces of the same coin, both caused by the severe immune system impairment affecting these patients? Can CMV favor IFD also in an early phase, through the alteration of immune surveillance and prior to its symptomatic manifestation? On the other hand, can we hypothesize that the fungal infection per se represent a risk factor for post-transplant CMV symptomatic reactivation in autologous setting? Should we consider the usefulness of an antifungal prophylaxis in patients with higher risk of CMV reactivation or, conversely, the need of a prospective surveillance of CMV DNAemia in patients at higher risk of IFD? At the best of our present knowledge, considering the different timing of such infections (median of 14 days for IFD and 34 days for CMV symptomatic reactivation, respectively), might be reasonable to begin a prospective monitoring of CMV DNAemia, instead of a clinically-driven approach, in patients that develop an IFD post-ASCT. Despite at present our study cannot directly address these issues, it is noteworthy that a previously published study in clinical setting distinct from the allogeneic transplant, reported a significant relationship between CMV and invasive mold infections at univariate analysis [42].

## 4. Materials and Methods 

### 4.1. Patients 

A single institution, retrospective, cohort study was conducted on a total of 347 non CD34+ selected autografts performed at the Hematology and Stem Cell Transplant Unit of Regina Elena National Cancer Institute of Rome (Italy) in the period comprised between May 2004 and April 2018. In case of tandem ASCT, the second transplant was excluded from the analysis. Over the 347 patients, 188 were affected by non-Hodgkin’s and Hodgkin’s lymphoma, whereas the remaining 159 by multiple myeloma. The patients who underwent an ASCT for an acute leukemia in the same period were excluded from this analysis. Patient characteristics at transplant are described in detail in Table 1. All patients received the same anti-infectious prophylaxis. In particular, all patients received an antiviral prophylaxis with valaciclovir and anti-Pneumocystiis prophylaxis with cotrimoxazole given from the day of transplant until six months after intervention and an anti-bacterial prophylaxis with ciprofloxacin from the day of transplant until the resolution of severe neutropenia. No mold-active prophylaxis was routinely administered. All patients were observed for a period of six months from transplant, with the aim to properly evaluate the occurrence of bacterial, fungal and viral infectious complications, as well as TRM: all clinical events occurring during this period were promptly captured and registered into an electronic database. All patients had signed an informed consent for transplant, also granting the use of sensitive data for scientific purposes.

### 4.2. Criteria for Diagnosis of CMV Symptomatic Infection and End-Organ Disease 

The criteria were based on published recommendations [43,44,45,46,47]. According to local policy [13] and published guidelines [43,47], CMV DNAemia was determined only upon clinical suspicion of post-transplant reactivation, therefore no routine monitoring CMV strategy was adopted. Criteria of clinical suspicion and assessment of CMV DNAemia, were defined as follows: presence of fever (temperature >38 °C) and overt clinical signs of bone marrow suppression in the absence of concomitant bacterial, viral (i.e., HHV-6, EBV, parvovirus B19) or fungal co-infections. Bone marrow suppression was defined as a delay of neutrophils and/or platelet recovery from ASCT (absence of complete neutrophils and platelets recovery after 14 and 21 days from transplantation, respectively) or a drop in neutrophils and/or platelet count after recovery (absolute count of neutrophils or platelets < 1.000/mm^3^ or 100.000/mm^3^, respectively, or a decrease of at least 30% of the counts in two consecutive determinations). CMV symptomatic infection was defined as a documented CMV DNAemia, confirmed by two consecutive determinations, in the presence of clinical suspicion criteria of reactivation. CMV end-organ disease was defined by the presence of signs consistent with CMV infection, as determined by a combination of imaging and clinical and histopathological/molecular evaluations. In particular, CMV gastrointestinal disease was defined by the presence of a combination of clinical symptoms from the upper or lower gastrointestinal tract, findings of macroscopic mucosal lesions on endoscopy, and demonstration of the presence of CMV inclusion bodies in the tissue biopsy, further confirmed by positive immunohistochemical staining of CMV antigens in tissue sections of the gastrointestinal tract. CMV pneumonia was defined by the presence of clinical (hypoxemia) and radiological signs of interstitial pulmonary disease, combined with the detection of high CMV viral loads by quantitative PCR in bronchoalveolar lavage fluid and confirmed by the detection of CMV by direct immunostaining of alveolar cells [44,46]. Lung tissue biopsies to demonstrate the presence of CMV inclusion bodies in the tissue biopsy were not performed, considering the high risk of complications derived from a pulmonary biopsy in patients with a severe respiratory distress and a great hemorrhagic risk. In the presence of signs and symptoms of CMV reactivation, as above specified, an antiviral treatment was administered. The choice of the antiviral agent to treat a symptomatic reactivationwas based on the clinical features of the patients at the time of reactivation.

### 4.3. Quantification of CMV DNA 

Automated nucleic acid sample preparation systems NucliSENSeasyMAG^®^ (BioMerieux, Durham, USA) has been used for DNA extraction from plasma, according to the manufacturer’s instructions. Amplification for detection and quantification of viral DNA has been performed using commercially available real-time PCR assays (Affigene^®^ CMV Trender diagnostic assay, Cepheid AB, Bromma, Sweden), according to the manufacturer’s instructions (Cepheid AB, Bromma, Sweden) on a Mx3000P^®^ System (Stratagene, La Jolla, CA, USA) until August 2013 then the analogous Geneproof CMV PCR kit (GeneProof, Brno, Czech Republic.) on SLAN^®^ Real-Time PCR Detection System (Shanghai Hongshi Medical Technology Co., Ltd., Shanghai, China). The limit of detection was 88 copies/mL with both kits.

### 4.4. Criteria for Diagnosis of Invasive Fungal Infections 

Invasive fungal infections were defined according to criteria of the EORTC/MSG [48]. In particular, a probable diagnosis of aspergillosis required that a host factor (i.e., recent history of neutropenia), a clinical feature (i.e., typical radiological findings) and a mycological evidence (i.e., galactomannan antigen detected in plasma, serum, bronchoalveolar lavage fluid or cerebrum-spinal fluid) were present. On the other hand, a proven aspergillosis required microscopic or microbiological evidence from a sterile culture of *Aspergillus* strains. A candidemia was diagnosed on the basis of at least one blood culture positive for a *Candida* spp strain, associated to clinical symptoms consistent with fungal pathogens (i.e., fever, hypotension, respiratory distress, gastrointestinal symptoms, hepatic or spleen involvement). Candidemia was considered as CVC-related if the isolated strain grew from CVC drawn blood or from a removed CVC-tip [49]. In the presence of signs and symptoms of invasive fungal infection, as above specified, an antifungal treatment was performed. As for therapeutic algorithm, we adopted a clinically-driven strategy in case of fever or lung infiltrates during neutropenic phase, according to published indications [50], without any planned weekly surveillance of serological markers of IFD. The choice of the antifungal agent was based on local policy and international guidelines.

### 4.5. Galactomannan Detection

The diagnostic Galactomannan testing, in serum and bronchoalveolar lavage (BAL) fluid samples, was performed in a routine clinical setting by the Platelia Aspergillus enzyme immunoassay (Bio-Rad Laboratories, Marnes-la-Coquette, France), according to the manufacturer’s instructions. Galactomannan level was measured spectrophotometrically by using the automated PhD™ lx System (Bio-Rad Laboratories, Hercules, CA, USA). Serum and BAL samples with an optical density index ≤ 0.5, were considered negative for galactomannan antigen

### 4.6. Criteria for Diagnosis of Bacterial Infections

A BSI was defined by the isolation of a bacterium in at least one blood culture, in association with signs and symptoms of sepsis; two positive cultures were required for diagnosis of coagulase-negative staphylococci or *Corynebacterium* spp. BSI. Clinically documented infections were defined as infections clinically or radiologically documented in the absence of a microbiological determination (i.e., unspecified pneumonia, colitis, skin and soft tissues infections).

### 4.7. Statistical Analysis

Data were analyzed by Statistical Package of Social Sciences software (SPSS, version 20.0, Chicago, IL, USA). For statistical analysis we considered each transplant as a single event and patients undergoing a double transplant were considered as two different events. Univariate analysis was performed in order to identify risk factors for bacterial, CMV and fungal infections by using χ^2^ test (Fisher or Pearson) and analysis of variance for categorical and quantitative variables, respectively. Two-sided *p*-values below 0.05 were considered to be statistically significant for the multivariate analysis. In case of two or more significant variables with reciprocal competitive effect, only the variable statistically more significant or clinically more relevant was included in the final model. Binary logistic regression model was used to analyze the associations between significant risk factors and the occurrence of infections. Enter and remove limits were 0.05 and 0.1, respectively.

## 5. Conclusions

Our data, gathered from adult patients with lymphoproliferative malignancies undergoing ASCT indicate that (1) Myeloma patients are at lower risk of bacterial infections and IFD when compared with lymphoma patients, but are at equal risk of CMV symptomatic reactivation, likely as a consequence of a selective impact of bortezomib on Herpes Viruses infection. (2) a significant association exists in the autologous setting between CMV reactivation and IFD, though a cause-effect relationship between these post-transplant events remains to be determined; (3) Bacterial infections, in particular by gram negative microorganisms, are the most frequent infectious complications with an overall case fatality rate of 4%; (4) The overall incidence of post-transplant CMV symptomatic infection and end-organ disease is about 15% and 1% respectively, with a low fatality rate (3.7%). Among lymphoma patients a diagnosis of T-cell lymphoma is the only factor significantly associated with the risk of CMV reactivation; (5) The overall IFD incidence is of about 3.5% with a fatality rate of about 17%. Patients with lymphoma, age of 60 years or older and having received three or more previous treatment lines of chemotherapy are at higher risk of IFD at multivariate analysis. Although, these data need to be confirmed by further prospective studies, the results strongly indicate that clinicians should be aware that IFD is a rare complication after ASCT but burdened by a high mortality rate, frequently associated with a CMV reactivation, and more often observed in lymphoma patients with advanced age and a long previous treatment history.

## Figures and Tables

**Table 1:** Features of 347 ASCTs performed in myeloma and lymphoma patients in our institution between May 2004 and April 2018.

Demographic Features at Transplant	
Median Age, years (Range)	56 (18–72)
Age	
<60 years	230 (67%)
≥60 years	117 (33%)
Sex	
M	208 (60%)
F	139 (40%)
Diagnosis	
MM	159 (46%)
B NHL	110 (32%)
HL	46 (13%)
T NHL	32 (9%)
Previous treatment lines	
1	193 (56%)
2	111 (32%)
≥3	43 (12%)
Disease status at transplant	
CR	213 (61%)
PR	115 (33%)
SD/PD	19 (6%)
Conditioning regimen	
BEAM/FEAM	188 (54%)
MEL200/MEL140/MEL100	159 (46%)
Median CD34+ × 10^6^/Kg infused cells (range)	5.66 (2.36–28.48)
Hematological recovery, median days (range)	
Neutrophils > 500/mm^3^	11 (8–30)
Platelets > 20.000/mm^3^	13 (9–120)
Transplant period	
2004–2011	112 (32%)
2012–2018	235 (68%)

ASCT: autologous hematopoietic stem cell transplant; MM: multiple myeloma; NHL: non Hodgkin lymphoma; HL: Hodgkin’s lymphoma; CR: complete response; PR: partial response; SD: stable disease; PD: progressive disease; BEAM: Carmustine, Etoposide, Cytarabine, Melphalan; FEAM: Fotemustine, Etoposide, Cytarabine, Melphalan; MEL200: Melphalan 200 mg/m^2^; MEL140: Melphalan 140 mg/m^2^; MEL100: Melphalan 100 mg/m^2^.

**Table 2:** Infection rates in our cohort of hematologic patients undergoing ASCT.

Type of Infection	Lymphoma pts.(188)	Myeloma pts.(159)	*p*
Neutropenic fever without microbiologically and/or clinically documented infection	48 (25.5%)	12 (7.5%)	<0.001
Overall bacterial infections	80 (42.5%)	45 (28%)	0.005
Gram positive BSI	29 (15.4%)	16 (10%)	NS
Gram negative BSI	32 (17%)	15 (9.4%)	NS
Other infections (*)	19 (10%)	13 (8.2%)	NS
CMV symptomatic infections	30 (16%)	24 (15%)	NS
CMV end-organ diseases	4 (2.1%)	1 (0.6%)	NS
Overall fungal infections	10 (5.3%)	2 (1.2%)	0.035
Candidemia	3 (1.6%)	1 (0.6%)	NS
Probable pulmonary aspergillosis (**)	7 (3.7%)	1(0.6%)	0.036
TRM	8 (4.2%)	2 (1.2%)	NS
Causes of death			-
CMV	1	1	
Fungal infection	1	1	
Bacterial infection	5	-	
Non-infective causes	1	-	

BSI: bloodstream infections; TRM: transplant-related mortality; NS: not statistically significant. (*) Others: mixed BSI, unspecified pneumonia, colitis, skin and soft tissues infections and urinary tract infections are included into this category. (**) Definition according to EORTC/MSG 2008 criteria.

**Table 3:** Clinical and laboratory features and outcome of CMV reactivation episodes requiring specific antiviral treatment.

Clinical and Laboratory Features	No. of Cases (All pts.)	No. of Cases (Lymphoma pts.)	No. of Cases (Myeloma pts.)
Overall incidence (%)	54/347 (15.5%)	30/188 (16%)	24/159 (15%)
Fever (temperature >38 °C persistent at least 60 min)	54 (100%)	30 (100%)	24 (100%)
Signs of bone marrow suppression (delay of neutrophils and/or platelet recovery or drop in neutrophils and/or platelet count after recovery)	52 (96%)	29 (97%)	23 (96%)
End-organ disease (according to published criteria)	5 (9.2%)	4 (13%)	1 (4.2%)
Interstitial pneumonia	3	2	1
Enteritis	2	2	0
Median day from transplant at first detection (range)	34 (12–77)	34 (13–70)	34 (12–77)
**Outcome**			
Treatment (*)			
Ganciclovir	15	8	7
Foscarnet sodium	15	10	5
Valganciclovir	24	12	12
Polyclonal immunoglobulins	4	4	4
Need of hospital admission	23 (42.6%)	15 (50%)	8 (33.3%)
Hematological recovery, median days (range) (**)			
Neutrophils> 500/mm^3^	12 (9–22)	11 (9–19)	12 (9–22)
Platelets > 20.000/mm^3^	15 (11–94)	16 (11–53)	15 (11–94)
Alive	50 (92.6%)	27 (90%)	23 (96%)
Dead (***)	4 (7.4%)	3 (10%)	1 (4%)

(*) Foscarnet sodium dosage: 60 mg/Kg twice daily for 14 days, than 60 mg/Kg/day for subsequent 5 days weekly for 2 weeks); Ganciclovir dosage: 5 mg/Kg twice daily for 14 days, than 5 mg/Kg/day for subsequent 5 days weekly for 2 weeks; Valganciclovir dosage: 900 mg twice daily for 14 days, than 900 mg/day for subsequent 5 days weekly for 2 weeks; (**) The occurrence of a clinically relevant CMV reactivation after ASCT, requiring antiviral treatment, leads to a delay in neutrophils and platelets recovery (*p* = 0.003 and *p* = 0.001 respectively); (***) Dead at 33, 48, 62, 89 days from transplant.

**Table 4:** Risk factors for CMV, fungal and bacterial infection in our study cohort of 347 patients.

Risk Factors (Univariate Analysis)	Fungal Infections		Bacterial Infections		CMV Symptomatic Infection or End-Organ Disease	
	Yes	*p*	Yes	*p*	Yes	*p*
**Diagnosis**		0.035		0.005		NS
Lymphoma (#188)	10 (5.3%)		80 (42.5%)		30 (16%)	
Myeloma (#159)	2 (1.2%)		45 (28%)		24 (15%)	
**Disease status at transplant**		NS		0.04		0.039
CR + PR (#328)	11 (3.3%)		116 (35.4%)		47 (14.3%)	
Refractory disease (#19)	1 (5.3%)		9 (47.4%)		7 (36.8%)	
Neutropenia duration (*)		NS		<0.001		0.004
<7 days (#123)	4 (3.2%)		25 (20.3%)		10 (8.1%)	
≥7 days (#219)	8 (3.6%)		100 (45.6%)		44 (20%)	
**Previous treatment lines**		0.009		NS		NS
≤2 (#304)	7 (2.3%)		113 (37.2%)		48 (15.8%)	
3 or more (#43)	5 (11.6%)		12 (27.9%)		6 (13.9%)	
**Age**		0.001		NS		NS
<60 years (#230)	3 (1.8%)		84 (36.5%)		31 (13.5%)	
≥60 years (#117)	9 (7.7%)		41 (35%)		23 (19.6%)	
**Transplant period**		NS		NS		NS
2004–2011 (#112)	2 (1.8%)		44 (39.3%)		18 (16.1%)	
2012–2018 (#235)	10 (4.2%)		81 (34.5%)		36 (15.3%)	

CR: complete remission; PR: complete remission; CMV: cytomegalovirus; NS: not statistically significant. (*) data not available in 5 cases (death in aplasia).

**Table 5:** Multivariate analysis of risk factors for CMV, fungal and bacterial infections in our study cohort of 347 patients.

Features	OR	95%CI	*p*
Fungal infections			
Age (≥60 years vs. <60 years)	10.34	2.55–40.11	0.001
Previous treatment lines (3 or more vs. ≤2)	2.91	1.29–6.55	0.012
Diagnosis (lymphoma vs. myeloma)	4.09	1.2–16.23	0.039
Bacterial infections			
Disease status (CR-PR vs. refractory disease)	1.06	0.41–1.29	NS
ANC < 500/mm^3^ (≥7 vs. <7 days)	2.16	1.29–3.74	0.006
Diagnosis (lymphoma vs. myeloma)	1.23	0.72–2.04	NS
CMV symptomatic infection/end-organ disease			
ANC < 500/mm^3^ (≥7 vs. <7 days)	2.4	1.2–4.9	0.009
Disease status (CR-PR vs. refractory disease)	0.69	0.39–1.1	NS

CMV: Cytomegalovirus; CR: complete response; PR: partial response; ANC: absolute neutrophils count; OR: odd ratios; 95%CI: 95% confidence interval.

**Table 6:** Risk factors for CMV, fungal and bacterial infection in our study cohort of 188 ASCTs performed in lymphoma patients.

Risk Factors(Univariate Analysis)	Fungal Infections		Bacterial Infections		CMV Symptomatic Infection or End-Organ Disease	
	Yes	*p*	Yes	*p*	Yes	*p*
**Diagnosis**		NS		NS		0.028
HL (#51)	1 (2%)		15 (29.4%)		3 (5.9%)	
B-NHL (#106)	7 (6.6%)		49 (46.2%)		19 (17.9%)	
T-NHL (#31)	2 (6.5%)		16 (51.6%)		8 (26%)	
**Disease status at transplant**		NS		NS		NS
CR + PR (#177)	9 (5%)		76 (42.9%)		28 (15.8%)	
Refractory disease (#11)	1 (9.1%)		4 (36.4%)		2 (18.2%)	
Neutropenia duration (*)		NS		0.022		NS
<7 days (#7)	1 (14.3%)		0		0	
≥7 days (#177)	9 (5%)		78 (44%)		30 (16.9%)	
**Previous treatment lines**		0.031		NS		NS
≤2 (#158)	6 (3.8%)		68 (43%)		24 (15.2%)	
3 or more (#30)	4 (13.3%)		12 (40%)		6 (20%)	
**Age**		0.002		NS		NS
<60 years (#136)	3 (2.2%)		58 (42.6%)		18 (13.2%)	
≥60 years (#52)	7 (13.5%)		22 (42.3%)		12 (23.1%)	
**Multivariate Analysis for Fungal Infections**	**OR**		**95%CI**		***p***	
Previous treatment lines (3 or more vs. ≤2)	4.53		1.1–18.53		0.036	
Age (≥60 years vs.<60 years)	7.58		1.83–31.7		0.005	

HL: Hodgkin’s lymphoma; B-NHL: B-cell non-Hodgkin’s lymphoma; T-NHL: T-cell non-Hodgkin’s lymphoma; CR: complete remission; PR: complete remission; CMV: cytomegalovirus; NS: not statistically significant; OR: odd ratios; 95%CI: 95% confidence interval. (*) data not available in 4 cases (death in aplasia).

## Abbreviations

ASCT	Autologous hematopoietic stem cell transplant
CMV	Cytomegalovirus
IFD	Invasive fungal disease
OR	Odd ratios
CI	Confidence intervals
ANC	Absolute neutrophils count
TRM	Transplant-related mortality
MM	Multiple myeloma
NHL	Non Hodgkin’s lymphoma
HL	Hodgkin’s lymphoma
CR	Complete remission
PR	Partial remission
SD	Stable disease
PD	Progressive disease
BEAM	Carmustine, etoposide, cytarabine, melphalan
FEAM	Fotemustine, etoposide, cytarabine, melphalan
MEL200	Melphalan 200 mg/m^2^
MEL140	Melphalan 140 mg/m^2^
MEL100	Melphalan 100 mg/m^2^
BSI	Bloodstream infection
NS	Not statistically significant
PCR	Polymerase-chains-reaction
CVC	Central venous catheter
